# Rigorous and rapid evidence assessment in digital health with the evidence DEFINED framework

**DOI:** 10.1038/s41746-023-00836-5

**Published:** 2023-05-31

**Authors:** Jordan Silberman, Paul Wicks, Smit Patel, Siavash Sarlati, Siyeon Park, Igor O. Korolev, Jenna R. Carl, Jocelynn T. Owusu, Vimal Mishra, Manpreet Kaur, Vincent J. Willey, Madalina L. Sucala, Tim R. Campellone, Cindy Geoghegan, Isaac R. Rodriguez-Chavez, Benjamin Vandendriessche, The Evidence DEFINED Workgroup, Siyeon Park, Vimal Mishra, Isaac R. Rodriguez-Chavez, Jennifer C. Goldsack

**Affiliations:** 1 Office of Medical Policy and Technology Assessment, Elevance Health, Palo Alto, CA USA; 2 Wicks Digital Health, Lichfield, UK; 3 Digital Medicine Society, Boston, MA USA; 4 Department of Emergency Medicine, School of Medicine, University of California, San Francisco, CA USA; 5 Geisinger Health System, Danville, PA USA; 6 UConn Health, Farmington, CT USA; 7 Big Health Inc., San Francisco, CA USA; 8 Lyra Health, Burlingame, CA USA; 9 Department of Medicine and Health Administration, Virginia Commonwealth University, Richmond, VA USA; 10 HealthCore, Inc., Wilmington, DE USA; 11 AstraZeneca, Inc., NY, NY USA; 12 Click Therapeutics, New York, NY USA; 13 Patients and Partners, LLC, Madison, CT USA; 14 ICON plc, Blue Bell, PA USA; 15 Department of Electrical, Computer and Systems Engineering, Case Western Reserve University, Cleveland, OH USA; 16 Byteflies, Antwerp, Belgium; 17 Present Address: Pharmesol, Inc., Newton, MA USA; 18 Present Address: UC Davis Health, Sacramento, CA USA; 19 Present Address: 4Biosolutions Consulting, Rockville, MD USA

**Keywords:** Therapeutics, Lifestyle modification, Clinical trials, Outcomes research, Translational research

## Abstract

Dozens of frameworks have been proposed to assess evidence for digital health interventions (DHIs), but existing frameworks may not facilitate DHI evidence reviews that meet the needs of stakeholder organizations including payers, health systems, trade organizations, and others. These organizations may benefit from a DHI assessment framework that is both rigorous and rapid. Here we propose a framework to assess Evidence in Digital health for EFfectiveness of INterventions with Evaluative Depth (Evidence DEFINED). Designed for real-world use, the Evidence DEFINED Quick Start Guide may help streamline DHI assessment. A checklist is provided summarizing high-priority evidence considerations in digital health. Evidence-to-recommendation guidelines are proposed, specifying degrees of adoption that may be appropriate for a range of evidence quality levels. Evidence DEFINED differs from prior frameworks in its inclusion of unique elements designed for rigor and speed. Rigor is increased by addressing three gaps in prior frameworks. First, prior frameworks are not adapted adequately to address evidence considerations that are unique to digital health. Second, prior frameworks do not specify evidence quality criteria requiring increased vigilance for DHIs in the current regulatory context. Third, extant frameworks rarely leverage established, robust methodologies that were developed for non-digital interventions. Speed is achieved in the Evidence DEFINED Framework through screening optimization and deprioritization of steps that may have limited value. The primary goals of Evidence DEFINED are to a) facilitate standardized, rapid, rigorous DHI evidence assessment in organizations and b) guide digital health solutions providers who wish to generate evidence that drives DHI adoption.

## Introduction

Digital health (DH) has proliferated in recent years^1,2^, with >300,000 health apps and over 300 wearables now available^1^. Organizations like the American Medical Association^3^ and American Psychiatric Association^4^ encourage digital health adoption, and more than half of U.S. adults use DH to track their health^5^. While digital health holds promise, current practices in DH have been described as the “Wild West”^6^, with misleading claims being common^7–9^, and clinical evidence quality often poor^2,7,10–14^.

Building on prior work^15^, we define digital health interventions (DHIs) as digital technologies intended to improve health outcomes and change health behaviors. Digital health interventions include products within the digital health, digital medicine, and digital therapeutic categories (see Table 1 for details). DHIs are often implemented using smartphone apps, wearables, and other technologies. Regulators have had a limited role in evaluating DHIs^7,13^, though this may change due to new functional areas within regulatory agencies (e.g., the FDA’s Digital Health Center of Excellence)^16,17^.

**Table 1:** Criteria defining digital health interventions (DHIs).

Criterion		
1. The product falls into one of the three classes of digital health technologies that were defined in a collaboration^15^ of stakeholders representing digital health trade organizations.	Product Class	Product Class Definition
	Digital Health	“Digital health includes technologies, platforms, and systems that engage consumers for lifestyle, wellness, and health-related purposes; capture, store or transmit health data; and/or support life science and clinical operations”^15^.
	Digital Medicine	“Digital medicine includes evidence-based software and/or hardware products that measure and/or intervene in the service of human health”^15^.
	Digital Therapeutics	“Digital therapeutic (DTx) products deliver evidence-based therapeutic intervention to prevent, manage, or treat a medical disorder or disease”^15^.
2. The product is designed to change one or more health behaviors.		
3. The value of the product to the evaluator is contingent on the degree to which it improves one or more health outcomes. These can include clinical outcomes (e.g., incidence of diabetic retinopathy) or surrogate outcomes (e.g., HbA_1C_).		

Following others^15,33^, we define digital health interventions as patient-facing products that meet the three criteria shown. DHIs are often implemented using smartphone apps, web platforms, consumer-grade wearables, and other digital technologies.

Following a preliminary search to identify existing frameworks for DHI evidence assessment (78 frameworks identified; see Supplementary Table 1), no framework was identified that met the needs of specific types of stakeholder organizations. The organizations that may benefit from an improved DHI assessment framework include payers, pharmacy benefit managers (PBMs), health systems, pharmaceutical companies, trade organizations, and professional medical societies. Throughout this article, the term *stakeholder organizations* refers to these organization types. Such organizations may benefit from a framework that is rigorous enough to identify clinically valuable DHIs reliably, yet rapid enough to accommodate the fast pace at which new DHIs enter the market.

Critical gaps (detailed below) were identified in extant DHI assessment frameworks, making them poorly suited for rigorous and rapid evaluation of clinical evidence. A multidisciplinary workgroup of leading experts was assembled to develop a careful and efficient strategy for DHI evidence evaluation in stakeholder organizations. The workgroup developed a novel framework to assess Evidence in Digital health for EFfectiveness of INterventions with Evaluative Depth (Evidence DEFINED). The Evidence DEFINED Framework builds on extant approaches, but differs in its inclusion of unique elements that are designed to increase rigor and speed. Efficiency in DHI assessment is critical given the ballooning number of DH technologies available^1,2^.

**Fig. 1:**
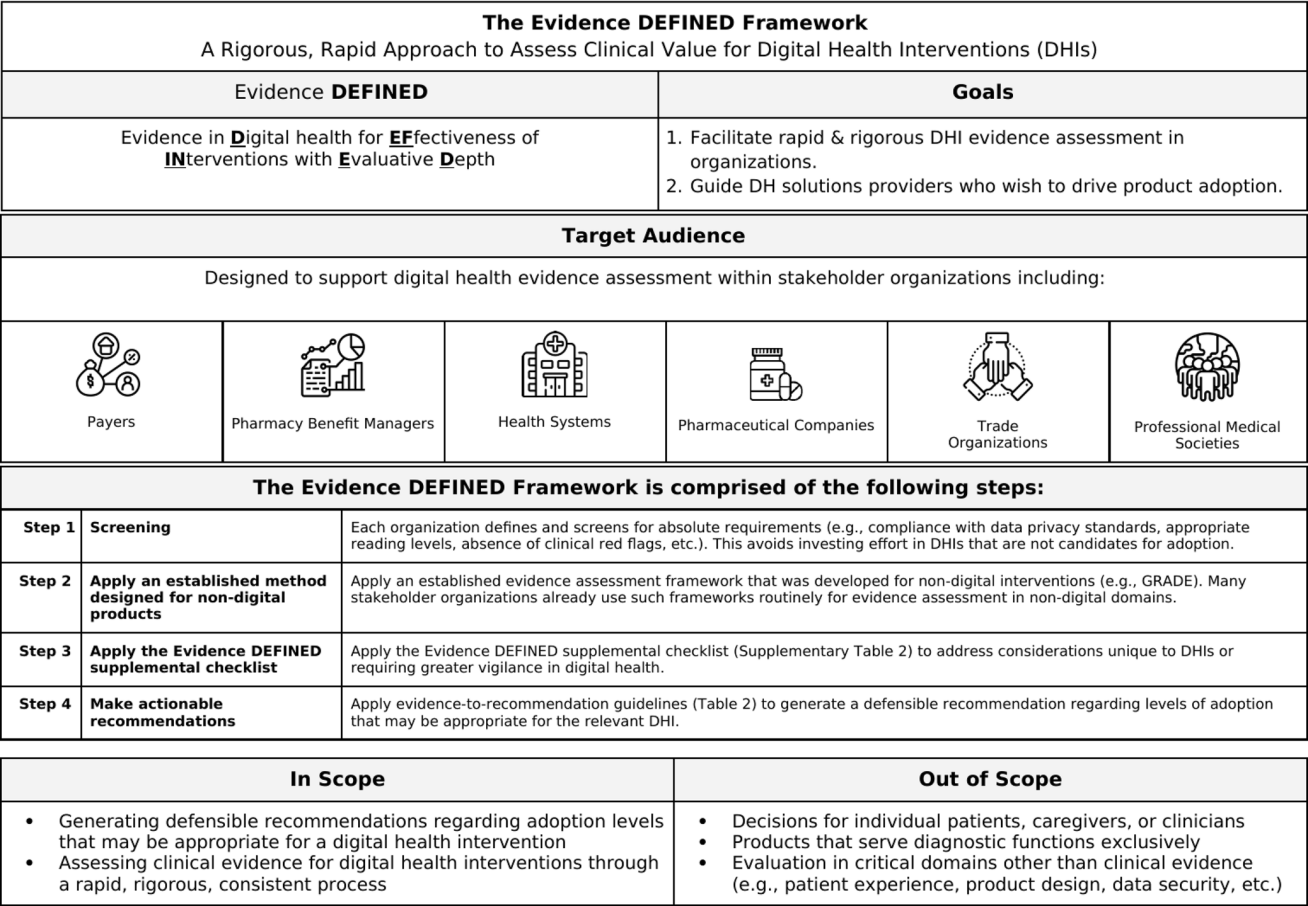
Quick start guide. A process overview for the Evidence DEFINED Framework.

Evidence DEFINED is a digital health evidence assessment process comprised of four steps, which are outlined in a Quick Start Guide (Fig. 1). The steps are (1) screen for failure to meet absolute requirements (e.g., compliance with data privacy standards), (2) apply an established evidence assessment methodology that was developed for non-digital interventions (e.g., GRADE^18^), (3) apply the Evidence DEFINED supplementary checklist (Supplementary Table 2), and (4) use evidence-to-recommendation guidelines (Table 2) to provide a recommendation regarding adoption levels that may be appropriate for the relevant DHI.

**Table 2:** Evidence-to-recommendation guidelines.

Actionability level	Criteria	Adoption level that may be appropriate	Approx. enrollment that may be appropriate
0	One or more of the following: • Clear evidence of harm or ineffectiveness for the current DHI version • The DHI is not clinically appropriate, per advice of clinical subject matter experts. • The risk balance is unfavorable due to safety concerns, per subject matter experts. • There are unaddressed concerns regarding misleading or false claims.	Adoption not recommended.	N/A
1	All of the following: • Very low or low-quality evidence (per GRADE^41^ definitions; “very low” includes no evidence) • Low clinical risk or well-managed risk with appropriate clinical rationale • Plausibility of clinically meaningful impact relative to usual care (or an alternate, relevant comparator) OR noninferior clinical outcomes with plausible improvement in a domain such as access, health equity, user experience, or cost. Clinically meaningful impact is defined by an effect size magnitude at or above a minimal clinically important difference, as established in credible guidelines and/or peer-reviewed literature.	Feasibility pilot: focus is on enrollment, engagement, user experience, safety.	*N* ≤ ~ 100
2	All of the following: • Meets or exceeds all criteria for Actionability Level 1 • Low-to-moderate quality evidence (per GRADE^41^ definitions). Real-world evidence may be included. • No or minimal uncertainty (per GRADE^41^) around value to stakeholders (often patients and their families) • Acceptable or likely acceptable (per GRADE^41^) to stakeholders	Small clinical pilot: primary outcomes are clinical.	Up to several hundred.
3	All of the following: • Meets or exceeds all criteria for Actionability Levels 1-2 • Moderate-to-high quality evidence (per GRADE^41^). Real-world evidence may be included.	Large clinical pilot: primary outcomes are clinical.	~300 ≤ *N* ≤ ~ 3000
4	All of the following: • Meets or exceeds all criteria for Actionability Levels 1–3 • Two or more high-quality RCTs support efficacy and safety • Preferred: one or more RCTs have 3rd-party data monitoring and analysis • Preferred: real-world evidence of safety and effectiveness	May be appropriate to scale.	No limit for appropriate patients.

These guidelines suggest degrees to which adoption of digital health interventions (DHIs) may be warranted by clinical evidence. Evidence is one of many critical assessment domains; others include patient experience, cost, health equity, etc. DHIs should be screened for failure to meet absolute requirements (e.g., HIPAA compliance). Enrollment targets are guidelines and should have statistical justification. For conditions with few treatments or urgent need, consider increasing rating by 1-2 actionability levels, without exceeding Level 3.

*DHI* Digital Health Intervention, *RCT* Randomized Controlled Trial, *HIPAA* Health Insurance Portability and Accountability Act.

The Evidence DEFINED framework has two primary goals. First, it will facilitate rigorous and rapid DH evidence assessment within the stakeholder organizations listed in Fig. 1, and thereby encourage adoption of DHIs that are most likely to improve health outcomes. Second, Evidence DEFINED will provide guidance to digital health solutions providers (DHSPs) who wish to generate evidence that drives adoption of their products. This may allow DHSPs to launch high-quality clinical trials with greater confidence that the investment is worthwhile. With clear and aligned evidence standards, DHSPs may face less uncertainty regarding the return on investment of clinical research.

## The need to assess evidence for digital health interventions

There is an urgent need to improve health outcomes and reduce costs, particularly for chronic conditions like diabetes, hypertension, depression, and many others^19–21^. Given the high prevalence of these conditions^22^, patient-centric and scalable solutions are needed to support condition self-management. Digital health interventions are one promising approach to help address this challenge^23^. But to realize that potential, only DHIs that are equitable, effective, and safe should be adopted^10,14^.

DHI adoption within the aforementioned types of organizations may sometimes be driven by marketing—not by evidence^14^. Criteria for DHI assessment often vary across and within stakeholder organizations. A “check the box” approach may be employed, where any DHSP presenting clinical outcomes may be deemed “validated”, whether or not this is an appropriate description, and irrespective of evidence quality. Where evidence quality is assessed, evaluative depth varies. Critical details may be overlooked, including risk for harm to patients. More rigorous and standardized evidence assessment methods are needed.

## Current evidence assessment frameworks

Dozens of frameworks have been proposed to assess evidentiary support for DHIs^13,24–28^. A comprehensive review is outside the scope of this paper, but a preliminary catalogue of frameworks investigated for this initiative is provided in Supplementary Table 1 (see also Supplementary Figure 1 and Supplementary Note 1). Seventy-eight prior frameworks were identified. Some of these may be useful, though many prior frameworks are underdeveloped in the key domain of clinical outcomes assessment^8^. Prior DHI evidence assessment frameworks are typically sections of broader DHI assessment guides, often containing just a few, superficial questions, with minimal evaluation of evidence quality or bias^29–32^.

To increase rigor in DHI evidence assessment, it may be helpful to address three gaps in prior frameworks. First, prior frameworks are not adapted to address evidence quality criteria that are unique to, or arise more commonly, in digital health interventions. For example, poor user experiences with digital health interventions can cause attrition of all but the most motivated patients. Such patients often show favorable outcomes, irrespective of any treatment effect. Thus, poorly-designed DHIs may sometimes retain only a biased subset of patients, who tend to show relatively strong outcomes. This may skew low-quality DHIs toward favorable clinical evaluations in per-protocol analyses of uncontrolled studies^33^. Such considerations may receive inadequate attention in routine DHI assessment.

Second, prior frameworks do not specify evidence quality criteria that may require increased vigilance given the current regulatory landscape in digital health. For example, in many cases, DHSPs may be nonadherent to trial registration and reporting best practices, which are detailed elsewhere^34^. This nonadherence is reflected in the 11% rate of public results reporting for registered DH trials^35^, despite the NIH recommending reporting within 12 months^36^. Even if some registered DH trials were within the 12-month reporting window at time of assessment, the low reporting percentage suggests that many negative results of DH trials may not be reported publicly, which could prevent appropriate evidence evaluation^34^. If a DHSP completed trials but did not report results publicly, this should impact assessments of evidence quality (see Supplementary Table 2 for specific recommendations). Adherence to other best practices should also be considered. Concerns around trial registration may be more common in DH, relative to therapeutic modalities (e.g., drugs) where trial registration is more regulated. Registration is one of many areas where increased vigilance regarding evidence quality may be appropriate for assessment of DHIs.

To address the first two gaps, Evidence DEFINED provides a supplementary checklist of evidence quality criteria that are recommended for DHI evidence assessments. This checklist (Supplementary Table 2) addresses evidence quality criteria that are unique to digital health, as well as evidence quality considerations that may require enhanced vigilance for assessment of digital health interventions.

The third gap in extant frameworks is that they fail to leverage established evidence evaluation methodologies that were developed for non-digital interventions (e.g., GRADE^18^). Such methodologies have undergone extensive development, with contributions from leading experts. Although many established evidence evaluation methodologies were designed for non-digital products (e.g., drugs), the principles pertain to DHIs. Rather than “reinventing the wheel”, the Evidence DEFINED framework utilizes established evidence assessment methodologies wherever possible.

## Scope of digital health products considered

A prior initiative, organized by the Digital Medicine Society (DiMe), developed a checklist to assess the evidence supporting fit-for-purpose biometric monitoring technologies (BioMeTs)^37^. Here we build on this work and develop a framework that may help organizational stakeholders assess digital health interventions. BioMeTs are out of scope, as are products that serve monitoring and diagnostic functions exclusively. The Evidence DEFINED Framework is not intended to support individual patient or clinician decisions; other frameworks (e.g., the App Evaluation Model of the American Psychiatric Association^4^) may be useful for this purpose.

We focus here on assessing clinical evidence for DHIs. Though out of scope for this initiative, other domains should also be evaluated. For example, DHI assessment should address patient experience, provider experience, product design, cost effectiveness, interoperability, etc. Data governance is a high-priority assessment domain, as inappropriate handling of health data can lead to serious patient harms^10^. DH equity is another critical domain; considerations may include language support, literacy, health literacy, digital literacy, numeracy, cultural appropriateness, and technical accessibility. Other frameworks have been proposed to assess these important domains^7,38–40^.

Note that DHIs may be in scope even if they are early in development and have yet to generate pivotal trial evidence. The potential value of young, innovative DH products should not be overlooked. Partnerships that help develop promising DH interventions should be encouraged, to spur needed innovation in healthcare. However, it is often appropriate to adjust adoption levels based on the maturity of a DHI’s clinical evidence. DHIs that have compelling evidence from high-quality trials may be appropriate to consider for widespread use, while those earlier in clinical development may be more appropriate to test in a limited number of patients. To guide DHI adoption levels that may be appropriate across varying levels of clinical evidence maturity, an evidence-to-recommendation framework is incorporated in Evidence DEFINED (Table 2). An actionability level is assigned, reflecting the degree to which clinical evidence may justify adoption of a digital health intervention.

## Rapid assessment

The aforementioned types of stakeholder organizations often require quick decisions to meet deadlines and move faster than competitors. Two key strategies are incorporated to achieve efficiency. First, the Framework uses screening items to determine whether a DHI meets absolute requirements. Assessment ends if the DHI fails to meet any absolute requirement. For example, time is not invested in evaluating evidence for a DH product that is not an adoption candidate due to non-compliance with privacy and security requirements. Second, as detailed below, a streamlined approach is used, avoiding information gathering that may have limited value.

## Evidence DEFINED implementation

Evidence DEFINED uses the following steps to facilitate rapid and rigorous evaluation of DHI evidence. We assume here that the DHIs under consideration have been identified. See Fig. 1 for a Quick Start Guide.

### Step 1. Screen for failure to meet absolute requirements

To avoid investing effort in DHIs that are not candidates for adoption, screen relevant DHIs for failure to meet absolute requirements. The screening step is applied flexibly; each stakeholder organization specifies their own requirements, per the organization’s needs. Screening requirements might include (a) a privacy policy that confirms compliance with HIPAA, (b) patient-facing language written at a targeted reading level (e.g., to comply with Medicaid guidelines), and (c) if subject to FDA regulation (detailed elsewhere^19^), the appropriate clearance or approval has been obtained. The screening step is similar to procedures recommended in the American Psychiatric Association’s App Evaluation Model^4^.

### Step 2. Apply an established evidence assessment framework

Apply an established evidence assessment framework that was developed for non-digital interventions (e.g., GRADE^41^). Many stakeholder organizations already use such frameworks routinely.

### Step 3. Apply the Evidence DEFINED supplementary checklist (Supplementary Table 2)

Apply the Evidence DEFINED supplementary checklist to address evidence quality considerations that are unique to digital health interventions, or that may require greater vigilance in digital health.

### Step 4. Make actionable, defensible recommendations

Apply evidence-to-recommendation guidelines (Table 2) to generate a recommendation around levels of adoption that may be appropriate. This guideline may help stakeholders generate defensible and actionable recommendations regarding appropriate adoption levels for digital health interventions.

These steps should be performed by evaluators with appropriate expertise, such as physicians, psychologists, pharmacists, researchers, clinical trialists, and biostatisticians. Organizations that do not have appropriate expertise internally may wish to partner with others. Any such partnerships should be conducted in an efficient manner. Organizations might consider service level agreements that specify assessment delivery dates.

## Exclusions from Evidence DEFINED

Evidence DEFINED is a streamlined framework. Many frameworks employ extensive feature lists^4,30,42,43^, and investigate which DHIs have which features. Such frameworks may be helpful where evidence is not available, and the goal is to determine which DHI is most likely to be effective and safe. A feature-focused approach may also be appropriate for a provider who seeks a digital health product meeting the needs of a specific patient. However, when applied to organizational decisions around DHI adoption, lengthy feature checklists may have at least two unfavorable consequences.

First, feature checklists can greatly increase the time required to evaluate DHIs. Using feature checklists in the evaluation process may require drafting feature lists and requesting information from digital health solutions providers. Cycles of information gathering often take months.

Second, feature checklists may yield misleading assessments of clinical value. Checking more boxes does not necessarily indicate that a DHI is effective and safe. Many DHSPs are sophisticated in their approach to requests for proposals (RFPs) and may prioritize “checking the box” over developing a feature that has genuine value. There is often a wide gap between the minimum level of effort required to claim defensibly that a product has a given feature, and the effort required to develop the feature to a degree that contributes meaningfully to improved clinical outcomes. It is common for DHSPs to develop “minimum viable product” (MVP) versions of a feature^44^. This may be appropriate, but evaluators should be aware of and adapt to this common practice in product development. In many cases, DHI features may be implemented at a level of refinement that permits “checking the box,” but does not provide clinical value.

If stakeholder organizations have a strong preference for specific features, then a small number of features can be assessed. We recommend, however, keeping feature checklists short. Assessments organized around feature lists may incent DH solutions providers to offer numerous, low-quality features, encouraging an unfavorable ratio of breadth to depth. Given these limitations, Evidence DEFINED focuses on evidence of safety and effectiveness—critical considerations to assess clinical value.

Note also that information sometimes gathered for DHSP assessment may have limited impact on decisions. Such information includes which venture capital firms fund the DHSP, the software development methods employed, corporate reporting structure, etc. Stakeholders should consider carefully how each piece of information will be used, and should consider foregoing information gathering that is unlikely to impact decisions.

## Updating the Evidence DEFINED Framework

Digital health is an evolving multidisciplinary field that itself is part of a large, complex healthcare ecosystem. Evidence DEFINED is agile and flexible to keep up with the pace of digital health innovation. As a leading professional organization in digital health, the Digital Medicine Society (DiMe) is an appropriate body to coordinate the updating process for the Evidence DEFINED Framework. Following others^37^, DiMe will establish a public website and collaborate with interested partners to update and disseminate the Evidence DEFINED Framework. The website will provide a suggestion form to gather input from the digital health community. Latest versions will be posted for the following Evidence DEFINED resources: the supplementary checklist of evidence quality criteria (Supplementary Table 2), evidence-to-recommendation guidelines (Table 2), and the Quick Start Guide (Fig. 1).

Given rapid evolution in digital health, Evidence DEFINED updates will be implemented every 6–12 months. Suggested modifications will be evaluated by article authors and other subject matter experts from the Society. Following a comment period, updated versions of the aforementioned key resources will be posted. See Supplementary Discussion for details.

## Development of the Evidence DEFINED Framework

Development of this Framework was organized by the Research Committee of the Digital Medicine Society, a nonprofit dedicated to advancing “safe, effective, equitable, and ethical use of digital medicine”^45^. The senior author (J.S.) facilitated the workgroup process and drafted initial materials, which were supplemented substantially and iterated upon by the multidisciplinary workgroup.

Seventeen experts with diverse backgrounds were assembled, representing academic medical centers, health plans, pharmaceutical companies, DH solutions providers, professional societies, patient advocacy organizations, and contract research organizations. Expertise within the workgroup spans clinical care, scientific research, biostatistics, health plan administration, regulatory affairs, and corporate strategy. Group members hold senior leadership positions in their organizations. A patient perspective representative (C.G.) was also included.

The workgroup agreed early in the process to develop a supplement–not a replacement–for established evidence assessment frameworks. Iterative feedback from workgroup members was solicited via asynchronous communications, four live workshops, and one-on-one discussions among workgroup members. The Evidence DEFINED Framework was refined based on edits and comments received during and following each live session. All group members provided feedback during at least one of the review cycles, and approved the final version.

## Discussion

Herein we have proposed the Evidence DEFINED Framework—a rigorous, rapid approach to assess the effectiveness and safety of digital health interventions. Evidence DEFINED may be appropriate for use by stakeholder organizations including payers, PBMs, health systems, pharmaceutical companies, trade organizations, and professional medical societies. The primary goal of the Evidence DEFINED Framework is to support high-quality, evidence-based decisions around adoption of digital health interventions, and thereby encourage use of safe and effective DHIs. Evidence DEFINED improves rigor by rectifying key gaps in prior approaches. The Framework achieves efficiency through screening steps and avoidance of information gathering that may have limited impact on decisions.

When assessing clinical evidence in digital health, details matter. Careful evidence assessment can mean the difference between identifying critical evidence flaws and failing to do so. This can, in turn, impact countless patients, by dictating whether patients get access to digital health interventions that are effective and safe. For some patients, rigorous DHI evidence assessment may mean the difference between medication adherence and nonadherence; between overcoming nicotine dependence and developing lung cancer; between resolution of affective symptoms and chronic emotional struggles. Because DHIs are scalable, relevant impacts may be magnified.

## Future directions

Best practices should be developed for coordinated, interdisciplinary DHI assessment, integrating well-developed methodologies across domains. Key assessment domains may include patient experience, provider experience, product design, cost effectiveness, data governance, interoperability, and health equity, as well as clinical evidence. Templates should be developed to summarize findings of Evidence DEFINED assessments and broader evaluations. The interrater reliability of Evidence DEFINED should be quantified in future research, and adjustments should be implemented if necessary. The Evidence DEFINED Checklist (Supplementary Table 2) may be adapted in the future for use in peer review.

Finally, best practices should be established that adapt trial design and statistical methods to accommodate the iterative nature of DHI development. Evidence DEFINED may facilitate initial assessments regarding appropriate adoption levels for a digital health intervention. More work is needed to establish best practices for monitoring post-trial DHI modifications (e.g., due to software updates), as well as any changes in safety or effectiveness, throughout the product lifecycle. Ultimately, DHI assessment will need to comply with an emerging regulatory framework, as well as quality assurance processes, to ensure consistency, appropriate evidence standards, and quality of the DHIs used by patients.

## Conclusions

To realize the potential of digital health, we need stronger, standardized frameworks for DHI evidence assessment^46^. We should encourage DH solutions providers to follow high standards—and hold DHSPs accountable to deliver the clinical value they promise. Evidence DEFINED may help guide DHSPs that wish to develop compelling evidence and drive adoption of digital health products.

Evidence DEFINED may also allow stakeholder organizations to assess DHI evidence in a more rapid, rigorous, and standardized manner. We hope this will promote evidence-based decision making, encourage adoption of effective DHIs, and thereby improve health outcomes across a range of conditions and populations.

## Methods

### Literature search overview

Scoping review methods^47^ were used to identify prior evidence assessment frameworks for digital health interventions (DHIs). A scoping approach was consistent with our goal to generate a preliminary assessment of relevant literature and its gaps^48^. Evidence assessment frameworks were identified from (a) 4 prior reviews^25–27,49^, (b) updating of MEDLINE searches performed for these reviews (to be current through October, 2022) and (c) a grey literature search performed per best practices detailed elsewhere^50^ (see Supplementary Figure 1). Due to differences in review scope, prior reviews included some assessment frameworks that did not address clinical evidence; such frameworks were excluded from this search. Following others^27^, we did not aim for and are unable to guarantee an exhaustive search, given the dynamic nature of this literature.

### Objectives of literature search

A literature search was performed with the objectives to (a) generate a preliminary list of relevant frameworks proposed previously, (b) provide a preliminary assessment regarding key characteristics of prior frameworks, and (c) assess the degree to which prior frameworks meet criteria that the Workgroup believed may facilitate rigorous and rapid assessment of digital health interventions. The criteria were (a) leveraging established evidence assessment methods that had been developed initially for non-digital interventions (e.g., GRADE^18^), (b) addressing evidence quality criteria that are specific to digital health interventions, (c) specifying evidence quality criteria that may require increased vigilance in digital health (given the current regulatory context), and (d) providing evidence-to-recommendation guidelines that state what levels of DHI adoption may be appropriate for varying degrees of evidence quality.

### Eligibility criteria

Frameworks were eligible for inclusion if they (a) were published in peer-reviewed or grey literature during or before October, 2022; (b) were described in one or more English documents; (c) recommended at least one criterion or question to assess evidentiary support for the safety, efficacy, or effectiveness of digital health interventions; (d) addressed clinical evidence either exclusively or in addition to other assessment domains (e.g., user experience, data security, etc.); and (e) were intended for application to either DHIs broadly or to a subgroup of DHIs (e.g., mental health apps). Frameworks were excluded that (a) addressed quality of health information but not evidence of safety/efficacy/effectiveness or b) were proprietary frameworks with minimal description of methods available publicly.

### Information sources and search strategy

Search strategies and information sources utilized in prior reviews are described elsewhere^25–27,49^. MEDLINE updates of prior searches were performed, to be current through October, 2022. Search strategies used for updating were the same as those described in the prior reviews^25–27,49^. Sources used for grey literature are detailed elsewhere^50^. These include Google Scholar as well as the websites of health technology assessment organizations, government agencies, and trade associations.

### Reporting summary

Further information on research design is available in the Nature Research Reporting Summary linked to this article.

## Supplementary information

Supplementary Material

Reporting Summary

## Data Availability

Supplementary Table 1 contains the only data that were collected for this manuscript.

## Code availability

No code was generated for this manuscript.
