# Supplementary material for: Rigorous and rapid evidence assessment in digital health with the evidence DEFINED framework
Source: NPJ Digit Med. 2023 May 31;6:101. doi: 10.1038/s41746-023-00836-5 (PMC10232404; doi:10.1038/s41746-023-00836-5)
Supplement: Supplementary file 1 — Supplementary Material [file 41746_2023_836_MOESM1_ESM.pdf]

SUPPLEMENTARY MATERIALS

Rigorous and Rapid Evidence Assessment in Digital Health with the Evidence DEFINED Framework

Jordan Silberman[1], MD, PhD; Paul Wicks[2], PhD; Smit Patel[3], PharmD; Siavash Sarlati[1,4], MD; Siyeon Park[5], PharmD; Igor O. Korolev[6], DO, PhD; Jenna R Carl[7], PhD; Jocelynn T. Owusu[8], PhD; Vimal Mishra[9], MD; Manpreet Kaur[1], MA; Vincent J. Willey[10], PharmD; Madalina L. Sucala[11], PhD; Tim R. Campellone[12], PhD; Cindy Geoghegan[3,13], BA; Isaac R. Rodriguez-Chavez[14], MSc, MHSc, PhD; Benjamin Vandendriessche[15,16], PhD; The Evidence DEFINED Workgroup* and Jennifer C. Goldsack[3], MChem, MA, MBA

[1]Office of Medical Policy and Technology Assessment, Elevance Health, Palo Alto, CA, USA
[2]Wicks Digital Health, Lichfield, United Kingdom
[3]Digital Medicine Society, Boston, MA, USA
[4]Department of Emergency Medicine, School of Medicine, University of California, San Francisco, CA, USA
[5]Geisinger Health System, Danville, PA, USA (former); Pharmesol, Inc., Newton, MA, USA (current)
[6]UConn Health, Farmington, CT, USA
[7]Big Health Inc., San Francisco, CA, USA
[8]Lyra Health, Burlingame, CA, USA
[9]Department of Medicine and Health Administration, Virginia Commonwealth University, Richmond, VA, USA (former); UC Davis Health, Sacramento, CA, USA (current)
[10]HealthCore, Inc., Wilmington, DE
[11]AstraZeneca, Inc., NY, NY
[12]Click Therapeutics, New York, NY, USA
[13]Patients and Partners, LLC, Madison, CT, USA
[14]ICON plc, Blue Bell, PA, USA
[15]Department of Electrical, Computer and Systems Engineering, Case Western Reserve University, Cleveland, OH, USA
[16]Byteflies, Antwerp, Belgium
*A list of authors and their affiliations appears at the end of the paper.

<u>Address correspondence to:</u>
Jordan M. Silberman, MD, PhD
Office of Medical Policy and Technology Assessment, Elevance Health
661 Bryant St.; Palo Alto, CA 94301
Phone: 650-294-0852
E-mail: jordan.silberman@elevancehealth.com

List of Supplementary Materials

1. **Supplementary Table 1**
2. **Supplementary Table 2**
3. **Supplementary Figure 1**
4. **Supplementary Note 1: Framework Counts**
5. **Supplementary Discussion**
6. **Supplementary References**

Supplementary Table 1. Evidence Assessment Frameworks for Digital Health Interventions. Frameworks address either evidence alone or evidence plus other domains of assessment. Framework criteria are as follows. A. Leverages established evidence assessment methods that were developed for non-digital interventions (eg, GRADE[1]). B. Addresses evidence quality criteria specific to digital health interventions (See Supplementary Table 2 for details). C. Specifies evidence quality criteria requiring increased vigilance for digital health interventions (See Supplementary Table 2 for details). D. Provides evidence-to-recommendation guidelines.

	Evidence Assessment Framework for Digital Health Interventions	Year	Framework Criteria				Addresses Non-Evidence Domains	Notes
			Criterion A	Criterion B	Criterion C	Criterion D		
1	6-Step Process for Evaluating Clinical Impact[2]	2022	N	N	N	N	Y	
2	Digital Therapeutics Alliance "Setting the Stage" recommendations[3]	2022	N	Y	N	N	Y	This document provides information on evidence generation strategy for DHIs. It is described as a “setting the stage” document; subsequent guidance may satisfy additional criteria.
3	Clinical robustness score[4]	2022	N	N	N	N	Y	Authors note that the evidence assessment component of this framework may be cursory. Focuses on FDA regulatory successes and entries on clinicaltrials.gov.
4	Multidimensional assessment framework for mobile medical apps.[5]	2022	N	Y	N	N	Y	Also referred to as "The DECALOGUE."
5	Medical Digital Solution Scoring Tool[6]	2022	N	N	N	N	Y	The evidence assessment component of this framework may be superficial.
6	Mental Health Apps Assessment Model, Anxiety and Depression Association of America[7]	2022	N	N	N	N	Y	Assesses for credibility, UX, data transparency
7	Digital Health Assessment Framework, ORCHA (updated 2022)[8]	2022	N	N	N	N	Y	Four assessment domains: data and privacy, clinical assurance and safety, usability and accessibility, technical security and stability. Evidence assessment approach is based on the NICE Evidence Standards Framework, which itself was introduced in 2019.

8	DTx Value Assessment and Integration Guide 2.0, Digital Therapeutics Alliance[9]	2022	Y	N	N	N	Y	Evidence quality statements were updated from an original draft. Some appear to be taken from GRADE assessment criteria but rephrased such that it may not be entirely clear what is being communicated. The evidence assessment component of this framework may be superficial.
9	App Rating Inventory Checklist[10]	2022	N	N	N	N	Y	
10	Framework to Assist Stakeholders in Technology Evaluation for Recovery (FASTER) to Mental Health and Wellness, Agency for Healthcare Research and Quality[11]	2022	N	N	N	N	Y	Incorporates screening to increase efficiency. The evidence assessment component of this framework may be superficial.
11	Review, Assess, Classify, and Evaluate (RACE)[12]	2022	N	N	N	N	Y	Focuses on assessing apps targeting opioid use disorder. Authors recommend that an RCT be conducted, though no further evidence assessment recommendations are provided.
12	Adapted checklist for assessment of mobile apps, National Library of Medicine, NIH.[13,14]	2022	N	N	N	N	Y	
13	AppScript Score (IQVIA)[15,16]	2021	N	N	N	N	N	The evidence assessment component of this framework may be superficial. Score based simply on study count, design type, and outcome of (published) studies. Evidence quality not addressed.
14	Return on health: Moving beyond dollars and cents in realizing the value of virtual care, American Medical Association[17]	2021	N	N	N	N	Y	
15	Review of criteria used to evaluate health apps.[18]	2021	N	N	N	N	Y	Meta framework.

16	ISO/TS 82304-2:2021(en) Health software — Part 2: Health and wellness apps—Quality and reliability[19]	2021	N	N	N	N	Y	
17	Adapted Mobile Application Rating Scale (A-MARS)[20]	2021	N	N	N	N	Y	Adaptation of Mobile Application Rating Scale (MARS) for mobile health apps plus other digital health technologies. The evidence assessment component of this framework may be superficial.
18	App Quality Assessment Scoring System (AQASS)[21]	2021	N	N	N	N	Y	Adaptation of Mobile Application Rating Scale (MARS). Focused on prenatal genetic testing. The evidence assessment component of this framework may be superficial.
19	Critical Outcomes Report Analysis, Validation Institute[22]	2021	N	N	Somewhat	N	N	This framework eschews peer review. Digital health solutions providers pay to be evaluated by this method.
20	Value-Drive Framework for Evaluating Healthcare Innovations, U.S. Department of Veterans Affairs[23]	2021	N	N	N	N	Y	Effectiveness criteria may be unclear.
21	ACCU3RATE[24]	2021	N	N	N	N	Y	Attempts to automate clinical evidence assessment using user reviews and developer product descriptions.
22	Digital Technology Assessment Criteria (DTAC), NHSx.[25]	2021	N	N	N	N	Y	
23	Mhealth Index and Navigation Database (MIND)[26]	2021	N	N	N	N	Y	Includes more than 100 questions. Evidence assessment questions address items like the count of studies and impact factor of any journals in which trials are published. The evidence assessment component of this framework may be superficial.
24	Digital Health Indicator, Healthcare Information and Management Systems Society (HIMSS)[27]	2020	N	N	N	N	Y	Pertains more broadly to a digital health ecosystem, which may include digital health interventions.

25	Benefit-Risk and Value Assessment Guide for Digital Health, Perakslis and Ginsburg, 2020[28]	2020	N	N	N	N	Y	Detailed in article supplement. Suggests a set of relevant assessment criteria.
26	App Quality Assessment Tool (AQUA)[29]	2020	N	N	N	N	Y	The evidence assessment component of this framework may be superficial.
27	THESIS Mobile Health Application Rating Tool[30]	2020	N	N	N	N	Y	The evidence assessment component of this framework may be superficial.
28	Unnamed guide to eHealth intervention assessment.[31]	2020	Y	N	N	N	Y	Incorporates elements of other frameworks
29	Technology Evaluation and Assessment Criteria for Health Apps (TEACH-Apps)[32]	2020	N	N	N	N	Y	Borrows heavily from the American Psychiatric Association App Evaluation Model.
30	A Framework for Evaluation of Mobile Apps for Youth Mental Health[33]	2020	Y	Somewhat	N	N	Y	The evidence assessment component of this framework may be superficial.
31	Digital Health Applications Ordinance - DiGA, Federal Ministry of Health, Germany.[34]	2020	Y	N	N	Y	Y	Encourages reporting of results no more than 12 months after study completion. Allows non-peer-reviewed publication. Requires trial registration and CONSORT reporting. At least a retrospective case-control study is required; higher levels of evidence are welcome. Evidence assessment practices may be inadequate in many cases, failing to account for the current "Wild West" state of Digital Health.
32	Unnamed framework for health technology assessment of mobile medical applications.[35]	2020	Y	Somewhat	N	N	Y	
33	Unnamed framework for selection of "credible" mHealth apps.[36]	2020	N	N	N	N	Y	The evidence assessment component of this framework may be superficial. Focuses on pediatrics populations.

34	Multi-attribute decision making framework for prioritization of mobile health applications[37]	2020	N	N	N	N	Y	
35	National Safety and Quality Digital Mental Health Standards, Australian Commission on Safety and Quality in HealthCare[38]	2020	N	N	N	Y	Y	
36	A decision-making checklist for selection of digital health technologies[39]	2020	N	N	N	N	Y	
37	A digital health assessment framework from an epidemiological and legal perspective[40]	2020	N	N	N	N	Y	
38	Unnamed set of questions for assessment of mHealth apps[41]	2020	N	N	N	N	Y	
39	Evidence Standards for Digital Health Technologies, National Institute for Health and Care Excellence (NICE)[42]	2019	N	N	N	Y	N	Evidence assessment practices may be inadequate in many cases, failing to account for the current "Wild West" state of Digital Health. See in particular the table starting on p. 17 of the NICE assessment guide.
40	App Evaluation Model, American Psychiatric Association[43]	2019	N	N	N	N	Y	Several alternate versions have been proposed.
41	Digital Assessment Questionnaire for Health Apps V2.2, NHS Digital.[44]	2019	N	N	N	N	Y	See Evidence of Outcomes section. This NHS framework asks the vendor to report whether there are any effectiveness claims. It may be possible to skirt

								requirements by stating a product is not designed to improve an outcome ("Lifestyle Loophole").
42	Xcertia mHealth App Guidelines[45]	2019	Y	N	N	N	Y	
43	Unnamed framework, Torous et al., 2019[46]	2019	N	N	N	N	Y	Refers to domains of evaluation, not details of an evaluation framework.
44	Digi-HTA[47]	2019	N	N	N	N	Y	
45	Digital Health Scorecard[48]	2019	Y	N	N	N	Y	
46	Design and Evaluation of Digital Health Interventions (DEDHI)[49]	2019	N	Y	N	N	Y	Focuses on assessment throughout the product lifecycle.
47	Unnamed framework for mHealth app assessment.[50]	2019	N	N	N	N	Y	This may be an iteration of the APA App Evaluation Model. Include screening steps for efficiency. Incorporates more clinical assessment questions than most. Limited assessment of evidence quality beyond asking basics about study design and impact factor of journals.
48	Transparency for Trust (T4T) Principles[51]	2019	N	N	N	N	Y	
49	Unnamed framework, Torous et al., 2018[52]	2018	N	N	N	N	Y	Overlaps with App Eval Model of the American Psychiatric Association.
50	Unnamed framework, Nouri 2018[53]	2018	N	N	N	N	Y	Review of mHealth app eval criteria. Far broader than clinical evidence. Authors consider clinical evidence a subdomain, not a priority. An adapted version[54] has been proposed for evaluation of apps targeting anxiety in pregnancy.
51	Mobile Health Practice Guide, US Department of Defense[55]	2018	N	Y	N	N	Y	
52	Toolkit for e-Mental Health Implementation, Mental Health	2018	N	N	N	N	Y	The evidence assessment component of this framework may be superficial.

	Commission of Canada[56]							
53	Assessment Framework for e-Mental Health Apps in Canada[57]	2018	N	N	N	N	Y	The evidence assessment component of this framework may be superficial.
54	Unnamed set of criteria for mHealth app assessment.[58]	2018	N	Y	N	N	Y	Key assessment criteria are listed in Box 3. The evidence assessment component of this framework may be superficial.
55	Framework for the Effectiveness Evaluation of Mobile (Mental) Health Tools, MindTech Healthcare Cooperative, National Institute for Health Research[59]	2017	Y	N	N	N	Y	
56	Enlight Evaluation Tool for Mobile and Web-Based eHealth Interventions[60]	2017	N	N	N	N	Y	The evidence assessment component of this framework may be superficial.
57	ORCHA-24 Framework[61]	2017	N	N	N	N	Y	The evidence assessment component of this framework may be superficial.
58	Set of heuristic methods for evaluation of digital health interventions.[62]	2017	N	Y	N	N	N	Proposes several thoughtful, heuristic, practical approaches to evidence assessment for digital health interventions. Screening approaches are addressed. Focuses on deployment of digital health interventions in low resource settings.
59	Pictorial identification scehma for diabetes self-care apps[63]	2016	N	N	N	N	Y	
60	Key questions for appraisal of digital health interventions.[64]	2016	N	N	N	N	N	See Table 1 in the cited article.
61	Mobile Health (mHealth) Evidence Reporting and	2016	Y	N	N	N	Y	From WHO mHealth Technical Evidence Review Group. Does not address DH-specific criteria. Focused on evidence reporting.

	Assessment (mERA) Checklist[65]							
62	Good Practice Guidelines on Health Apps and Smart Devices[66]	2016	N	N	N	N	Y	Evidence requirements are addressed but may be unclear.
63	Interactive Mobile App Review Toolkit (IMART)[67]	2016	N	N	N	N	Y	
64	Unnamed set of criteria for assessment of mobile medical applications in diabetes.[68]	2016	N	N	N	N	Y	
65	Mobile Application Rating Scale (MARS)[69]	2015	N	N	N	N	Y	The evidence assessment component of this framework may be superficial.
66	PAS 277:2015 - Innovate UK, British Standards Institution[70]	2015	N	N	N	N	Y	
67	Royal College of Physicians (RCP) Health Informatics Unit Checklist[71]	2015	N	N	N	N	Y	The evidence assessment component of this framework may be superficial.
68	Unnamed adaptation of NICE behavior change guidance for app quality assessment.[72]	2015	N	N	N	N	Y	See Appendix 2 for app quality assessment tool. Focuses on adherence to behavior change best practices, rather than evidence of effectiveness and safety.
69	Unnamed set of questions for assessment of mobile mental health apps.[73]	2015	N	N	N	N	Y	
70	Unnamed set of recommendations for assessment of mobile health apps.[74]	2014	N	N	N	N	Y	
71	Unnamed set of recommendations for	2014	N	N	N	N	Y	

	assessment of mobile health apps.[75]							
72	Evaluating Mobile Medical Applications, American Society of Hospital Pharmacists.[76]	2014	N	N	N	N	Y	
73	PsyberGuide[77]	2013	N	N	N	N	Y	
74	Synopsis for Health Apps.[78,79]	2013	N	N	N	N	Y	
75	Framework for assessment of mobile applications for cardiac rehab[80]	2013	N	N	N	Y	Y	Cursory framework reported as part of a broader article on cardiac rehab. Randomized controlled trials are encouraged. The evidence assessment component of this framework may be superficial.
76	Khoja–Durrani–Scott Evaluation Framework, PAN Asian Collaboration for Evidence-Based e-Health Adoption and Application[81]	2013	?	?	?	?	Y	Limited information available on framework details. Links to detailed framework instructions no longer work as of July 2022.
77	Model for Assessment of Telemedicine applications (MAST)[82]	2012	Y	Somewhat	N	N	Y	Encourages clinical evidence assessment. The evidence assessment component of this framework may be superficial.
78	CONSORT-eHealth Checklist Extension[83]	2011	Y	Somewhat	Somewhat	N	N	Checklist to assess manuscripts for peer review.

Abbreviation: PROs, Patient-Reported Outcomes

Supplementary Table 2. Checklist of Evidence Quality Criteria for Digital Health Interventions. This checklist is designed to supplement established evidence assessment frameworks. Group 1 Criteria are those where adaptations to established criteria are recommended, due to differences between digital and non-digital interventions. Group 2 Criteria pertain in both digital and non-digital domains, but increased vigilance is encouraged for DHIs in the current regulatory context.

	Evidence Assessment Criterion	Evidence Criterion Group	Rationale for Inclusion and Notes	Examples Meeting/ Not Meeting Criterion	Recommended Actionability Level (AL) Change if Not Met	Importance
1	DHI assessment is not based solely on association with eminent individuals or institutions.	Group 2. Increased vigilance recommended for DH.	Stakeholders may overvalue a DH solutions provider's association with eminent individuals or institutions. Though relevant, none of the following is a replacement for evidence:<br>- Expert advisors (may have no meaningful role)<br>- University collaboration (DHSPs can pay for this)<br>- KOL endorsements (they may not evaluate DHIs appropriately and often have conflicts of interest)<br>- Endorsement by compensated third parties<br>- Adoption by reputable clients (DHI assessment standards are highly variable, even across reputable organizations)	Example meeting criterion<br>High-quality, peer-reviewed evidence shows a mean A1c reduction of 0.7, relative to no change for randomly assigned control participants.<br>Example not meeting criterion<br>A KOL endorses a DHI's effectiveness based on unreviewed, low-quality evidence.	KOL endorsement should not impact AL rating.	Essential
2	Control condition is consistent with evaluator priorities.	Group 1. Adaptations recommended for DH.	Sham controls are designed to blind participants to trial arm assignment and equalize engagement across arms. This approach may allow unconfounded attribution of benefit to a DHI. However, "sham apps" may mask non-specific risks associated with increased smartphone exposure,[84] because smartphone use is equal across arms in trials employing sham apps. Growing evidence[85–90] suggests that increased smartphone exposure may harm mental health.<br>Usual care (UC) controls (defined elsewhere[91]) receive no treatment from the study. UC controlled trials cannot distinguish specific effects (eg, impact of app-delivered health education) from non-specific effects (eg, impact of taking time to use an app, which may reduce time exposed to stressors). However, UC control conditions should not mask the aforementioned non-specific harms, where they exist.<br>Advantages and disadvantages of other control condition types, including standard of care controls, are reviewed elsewhere.[91] Sham controls may be appropriate for explanatory trials, where DHI safety has been established. UC controls may be appropriate for pragmatic trials, where the goal is to generate evidence that guides real-world decisions, and where some non-specific mechanism of benefit is acceptable.	Examples meeting criterion<br>A high-quality trial with UC controls showed clinically and statistically significant benefit, and evaluators are comfortable with the possibility of non-specific mechanisms of benefit.<br>Example not meeting criterion<br>A high-quality trial with UC controls showed clinically and statistically significant benefit. Evaluators have stringent standards and want to know that benefits are mediated through specific mechanisms.	Decrease rating by 1 level if control condition is inconsistent with evaluator priorities	Essential for controlled studies

3	Results are not "cherry picked."	Group 2. Increased vigilance recommended for DH.	DHSPs may "cherry pick" analyses that show atypical effect sizes.<br><br>Note that a) for any intervention, different patient samples will show different effectiveness levels and b) some patient samples may show meaningful clinical benefit due to sampling error alone, even when the true effect size is zero.[92]<br><br>Risk for unrepresentativeness increases if studies are retrospective, unregistered, registered after start of enrollment, or small in sample size.	<u>Example meeting criterion</u><br><br>A preregistered, high-quality trial shows mean hemoglobin A1c reductions of 0.7, relative to no change observed for controls.<br><br><u>Example not meeting criterion</u><br><br>An unregistered, retrospective analysis excludes 90% of participants and reports robust clinical improvements among those retained.	Decrease rating by 1-2 levels.	Essential
4	Data missingness is addressed appropriately.	Group 2. Increased vigilance recommended for DH.	Substantial data missingness is common in DH.[93,94] It is often assumed implicitly that data are missing completely at random or at random (MCAR or MAR), even where these assumptions are implausible. This may cause underappreciated bias.<br><br>Missingness should be handled per best practices detailed elsewhere.[95–100] It is often appropriate to compare baseline scores by attrition status; meaningful differences rule out MCAR and MAR assumptions.[101] Sensitivity analyses should assess robustness of findings to "worst case"[95,102] and other[93,102] degrees of difference between missing and observed data.<br><br>Note that poor user experiences may cause attrition of all but the most motivated patients. If motivated patients have better outcomes on average, then this attrition pattern would paradoxically skew poorly designed DH products toward favorable per-protocol results.[84]	<u>Example meeting criterion</u><br><br>A trial reports 10% attrition, with statistically significant differences at baseline between completers and non-completers. However, sensitivity analyses reveal that study conclusions would hold under "worst case" assumptions.<br><br><u>Example not meeting criterion</u><br><br>A trial reports 40% attrition. No analyses address risk for biased missingness.	Decrease rating by 1-2 levels.	Essential
5	Intention-to-treat (ITT) analyses are reported and any per-protocol (PP) analyses are described as such.	Group 2. Increased vigilance recommended for DH.	ITT analyses should be reported wherever possible. Per-protocol (PP) analyses should be described as such, with reporting on the proportions of ALL enrolled participants who are included in each PP analysis.	<u>Example meeting criterion</u><br><br>ITT analyses show mean hemoglobin A1c reductions of 0.7, relative to no change observed for controls.<br><br><u>Example not meeting criterion</u><br><br>Only PP analyses are reported.	Decrease rating by 1-2 levels.	Essential

6	Trials are preregistered (eg, using clinicaltrials.gov) and results are reported publicly.	Group 2. Increased vigilance recommended for DH.	Trials should be registered prior to start of enrollment.[103] Results should be shared within 12 months of trial completion[104] wherever feasible, and should be published in peer-reviewed journals.<br><br>Registration is not required for some DHI commercialization paths. This can increase publication and reporting bias,[105] reducing replicability of findings. We therefore cannot predict that future DHI deployments will be as effective as reported in unregistered trials.<br><br>Note that all interventions show distributions of effect sizes across samples. Due to selective reporting and the inconsistency of trial registration in DH, many published DHI effect sizes may represent only the most favorable sliver of the relevant effect size distributions.	<u>Example meeting criterion</u><br><br>A preregistered, high-quality trial shows mean hemoglobin A1c reductions of 0.7, relative to no change for controls.<br><br><u>Example not meeting criterion</u><br><br>An unregistered trial shows robust reductions in hemoglobin A1c.	Decrease rating by 1-2 levels.	Essential
7	Conclusions regarding safety or effectiveness are not based on DHSP attestation alone.	Group 2. Increased vigilance recommended for DH.	Some digital health solutions providers (DHSPs) formally self-attest to following best practices, often in collaboration with a trade organization. This may be helpful, but self-attestation is not a substitute for evidence.	<u>Example meeting criterion</u><br><br>High-quality, peer-reviewed evidence shows a mean reduction in hemoglobin A1c of 0.7, relative to no change for controls.<br><br><u>Example not meeting criterion</u><br><br>A DHSP signed a self-attestation stating that they follow best practices.	Self-attestations should not impact ratings.	Essential
8	Marketing claims are consistent with peer-reviewed evidence and are not misleading.	Group 2. Increased vigilance recommended for DH.	In the current regulatory context, misleading and evidence-discordant claims are common.[28,48,106]	<u>Example meeting criterion</u><br><br>Reporting in a peer-reviewed article is consistent with marketing claims.<br><br><u>Example not meeting criterion</u><br><br>A DHSP changes patient-decades (in a peer-reviewed article) to patient-years (in marketing claims) without moving the decimal point, causing claims to be overstated by an order of magnitude.	Decrease rating by 1-2 levels.	Essential

9	Evidence is reported in peer-reviewed journals rather than white papers or other unreviewed materials.	Group 2. Increased vigilance recommended for DH.	Though peer review is often expected, some DHSPs rely on "white papers." These marketing documents may show levels of rigor and transparency that are inadequate for appropriate evidence assessment.<br><br>Evidence published in predatory journals (defined elsewhere[107]) is also inadequate.	<u>Example meeting criterion</u><br><br>High-quality, peer-reviewed evidence shows a mean reduction in hemoglobin A1c of 0.7, relative to no change for controls.<br><br><u>Example not meeting criterion</u><br><br>An uncontrolled, retrospective analysis for an unreported number of patients shows robust A1c reductions. Evidence is not peer-reviewed, but rather is reported in a white paper.	Non-peer-reviewed evidence can be considered, but alone does not justify any increase in actionability rating.<br><br>The same is true for evidence published in predatory journals.	Essential
10	Results are clinically and statistically plausible.	Group 2. Increased vigilance recommended for DH.	Implausible reporting does happen in digital health, even in high-impact, peer-reviewed journals.	<u>Example meeting criterion</u><br><br>All quantitative findings reported are plausible.<br><br><u>Example not meeting criterion</u><br><br>Reported confidence intervals imply a standard deviation of 45 for hemoglobin A1c, which is implausible clinically.	Decrease rating by 1-2 levels.	Essential
11	It is not assumed that numerous peer-reviewed publications indicate effectiveness or safety.	Group 2. Increased vigilance recommended for DH.	Published editorials may be relevant, but are not a substitute for evidence.<br><br>High numbers of published, low-quality studies should not be confused with high-quality evidence.	<u>Example meeting criterion</u><br><br>High-quality, peer-reviewed evidence shows a mean A1c reduction of 0.7, relative to no change in controls.<br><br><u>Example not meeting criterion</u><br><br>A DHSP published editorials but not clinical evidence.	Peer-reviewed editorials should not impact evidence ratings.<br><br>Low-quality evidence should not justify ALs greater than 2, even if multiple peer-reviewed articles are available.	Essential

12	Patients who declined to participate are not used as comparators.	Group 2. Increased vigilance recommended for DH.	Patients who enroll in health management programs often differ meaningfully from those who decline to participate.[108] For example, enrollees may have stronger motivation to self-manage chronic conditions. Matching on demographics does not resolve this.	<u>Example meeting criterion</u><br><br>The rate of acute clinical events for DHI users is 15% lower than that of randomly assigned, waitlisted controls.<br><br><u>Example not meeting criterion</u><br><br>The rate of acute clinical events for DHI users is 15% lower than that of demographics-matched adults who declined to participate.	Decrease rating by 1-2 levels.	Strongly Preferred
13	Observed clinical improvements are not attributable to healthy user effects or other selection biases.	Group 2. Increased vigilance recommended for DH.	Patients who use health management tools like DHIs may differ from those who do not.[108] DHI users may have stronger health-related motivations and exhibit healthier behaviors. Patients who use DHIs may show improved outcomes over time irrespective of intervention. It should not be assumed that clinical status would be static without intervention.<br><br>For example, a recent RCT[109] showed a meaningful 10.6 mm Hg reduction in systolic blood pressure for DHI users, but a comparable 10.1 mm Hg reduction for controls. Without a control arm, it would have been easy to misinterpret this as evidence of effectiveness.<br><br>In some cases it is possible to reduce risk of healthy user (and similar) biases. Investigators should follow best practices, summarized elsewhere,[108] to analyze and interpret data where healthy user (and other) biases may inflate effectiveness estimates.	<u>Example meeting criterion</u><br><br>High-quality, peer-reviewed evidence shows a mean A1c reduction of 0.7, relative to no change for randomly assigned controls.<br><br><u>Example not meeting criterion</u><br><br>An uncontrolled study shows a mean A1c reduction of 0.7.	Decrease rating by 1-2 levels.	Strongly Preferred where Relevant
14	Frequency and intensity of interaction with human personnel (eg, mental health professionals or health coaches) has not changed following evidence generation.	Group 1. Adaptations recommended for DH.	As DHI deployment scales up, or as business models evolve, intervention components previously implemented by program staff may be automated. Reducing human interaction may reduce effectiveness in some cases.[110]	<u>Example meeting criterion</u><br>High-quality evidence of efficacy was generated for an automated DHI product version.<br><br><u>Example not meeting criterion</u><br>High-quality, peer-reviewed evidence was generated for a DHI version incorporating video chat with a clinical pharmacist. After a pivotal trial, this intervention component was automated. No post-automation evidence is available.	Decrease rating by 1-2 levels, unless evidence shows noninferiority of an automated DHI product version, relative to a non-automated version.	Strongly Preferred

15	Qualifications of personnel delivering the intervention (eg, mental health professionals or health coaches) remain consistent following evidence generation.	Group 1. Adaptations recommended for DH.	As DHI use scales up, if more personnel are needed, minimum qualification requirements may be relaxed.	<u>Example meeting criterion</u><br><br>High-quality evidence was generated for a DHI product version after relaxing minimum qualifications required of DHI personnel.<br><br><u>Example not meeting criterion</u><br><br>High-quality evidence was generated for a DHI incorporating video chat with a clinical pharmacist. Subsequently, pharmacists were replaced with "care coordinators" who do not have clinical training. No evidence is available comparing DHI versions.	Decrease rating by 1 level.<br><br>An exception should be made if evidence shows noninferiority of a DHI version in which minimum qualifications of program personnel were relaxed.	Strongly Preferred
16	If the target population includes underserved patients, then study samples should have included such patients.	Group 1. Adaptations recommended for DH.	DHIs often require adaptations for underserved patient populations. For example, adaptations may be needed to address varying levels of literacy, health literacy, numeracy, digital literacy, and broadband access.	<u>Example meeting criterion</u><br><br>An organization is assessing a DHI for use in underserved patient communities. The DHI has shown effectiveness among racial minority subgroups as well as subgroups residing in low-SES zip codes.<br><br><u>Example not meeting criterion</u><br><br>An organization is assessing a DHI for use in underserved patient communities. Relevant studies investigated high-SES patients only.	Decrease rating by 1-2 levels.	Strongly Preferred

17	Effect sizes are comparable for registered and non-registered trials, if relevant.	Group 1. Adaptations recommended for DH.	This criterion pertains only to DHIs for which evidence has been generated in both registered and unregistered trials.<br><br>Trial registration (eg, through clinicaltrials.gov) is not required for some commercialization paths. This may increase publication bias[105] and reduce the likelihood of replicating reported effect sizes.<br><br>We do not expect any two studies to show identical effect sizes. But if effect sizes for registered and unregistered trials differ to a clinically meaningful degree, this may raise concern for publication bias.<br><br>Consider investigating differences across studies that may explain any effect size inconsistencies. Such differences may relate to DHI versions, implementation protocols, sample characteristics, or sample sizes (small samples increase risk for outlying effect sizes).	Example meeting criterion<br><br>High-quality, peer-reviewed evidence shows mean reductions in hemoglobin A1c of 0.7 and 0.5, both relative to no change observed for controls, in registered and unregistered trials, respectively.<br><br>Example not meeting criterion<br><br>High-quality, peer-reviewed evidence shows mean reductions in hemoglobin A1c of 0.7 and 0.1, both relative to no change observed for controls, in registered and unregistered trials, respectively.	Decrease rating by 1 level.	Strongly Preferred where Relevant
18	Distribution of effect sizes does not suggest meaningful uncertainty in average level of benefit.	Group 1. Adaptations recommended for DH.	For some non-digital treatment modalities (eg, drugs), effect size inconsistency may suggest uncertainty in average level of benefit. However, for DHIs, effect size inconsistency may be due to improvements implemented over time. Iterative improvement of DHIs is common and should not cause downgrading of evidence actionability.<br><br>Some evidence assessment frameworks designed for non-digital interventions recommend reducing ratings if effect sizes differ across studies of similar patient samples.[111] However, if effect sizes for a DHI improve over time, this may reflect product improvements.	Example meeting criterion<br><br>Mean A1c reductions of 0.4 and 0.8 were observed in high-quality studies conducted 4 and 2 years ago, respectively.<br><br>Example not meeting criterion<br><br>Mean A1c reductions of 0.8 and 0.4 were observed in high-quality studies conducted 4 and 2 years ago, respectively.	Do not reduce rating if effect size improves over time.<br><br>Decrease rating by 1-2 levels if unexplained, unfavorable changes in effect size are observed.	Strongly Preferred
19	DHI modifications implemented during and after trials are documented.	Group 1. Adaptations recommended for DH.	DHIs are often improved iteratively, through software updates. Current versions may have clinically meaningful differences from trialed versions.<br><br>DHSPs should report a) the product version in use at the start of a trial, b) the dates of product updates, and c) the product changes implemented with each update.	Example meeting criterion<br><br>Software versions used during and after a trial are reported in a public website. A summary of each update is provided.<br><br>Example not meeting criterion<br><br>Software versioning information is not reported.	Evaluators should be aware of this criterion, though AL adjustment may not be needed.	Preferred

20	Onboarding for trial participants is comparable to onboarding in real-world deployments.	Group 2. Increased vigilance recommended for DH.	Lengthy onboarding assessments are common in digital health trials. This may select for more motivated participants, on average. After DHI deployment, when enrollment may take precedence over evaluation, onboarding burden may be reduced substantially. On average, this may select for less motivated patients. Thus, average clinical benefit may be lower in real-world use.	Example meeting criterion<br><br>Onboarding assessments in a previous trial are the same as those used following real-world deployment.<br><br>Example not meeting criterion<br><br>Ten baseline PROs were administered in a pivotal trial, while two are administered following real-world deployment.	Evaluators should be aware of this criterion, though AL adjustment may not be needed.	Preferred
21	Participant incentives are comparable in trials and real-world deployments.	Group 2. Increased vigilance recommended for DH.	Trial participants may receive payment or other incentives for enrolling and meeting engagement targets. Changes in incentives between trials and subsequent real-world deployments may change who enrolls and how much they engage.<br><br>Stakeholders should be aware that this common scenario may decrease external validity of trial evidence.	Example meeting criterion<br><br>The same incentives are provided in a pivotal trial and a real-world deployment.<br><br>Example not meeting criterion<br><br>Trial participants are paid for high engagement; this payment is reduced in real-world DHI deployment.	Evaluators should be aware of this criterion, though AL adjustment may not be needed.	Preferred

Abbreviations: DH, Digital Health; DHIs, Digital Health Interventions; DHSPs, Digital Health Solutions Providers; AL, Actionability Level; KOL, Key Opinion Leader; MAR, Missing at Random; MCAR, Missing Completely at Random; PROs, Patient-Reported Outcomes; RCT, Randomized Controlled Trial; SES, Socioeconomic Status; UC, Usual Care

Supplementary Figure 1. PRISMA 2020 flow diagram. Prior reviews[26,49,53,112] and grey literature searching were used to identify relevant frameworks (see Methods for further details).

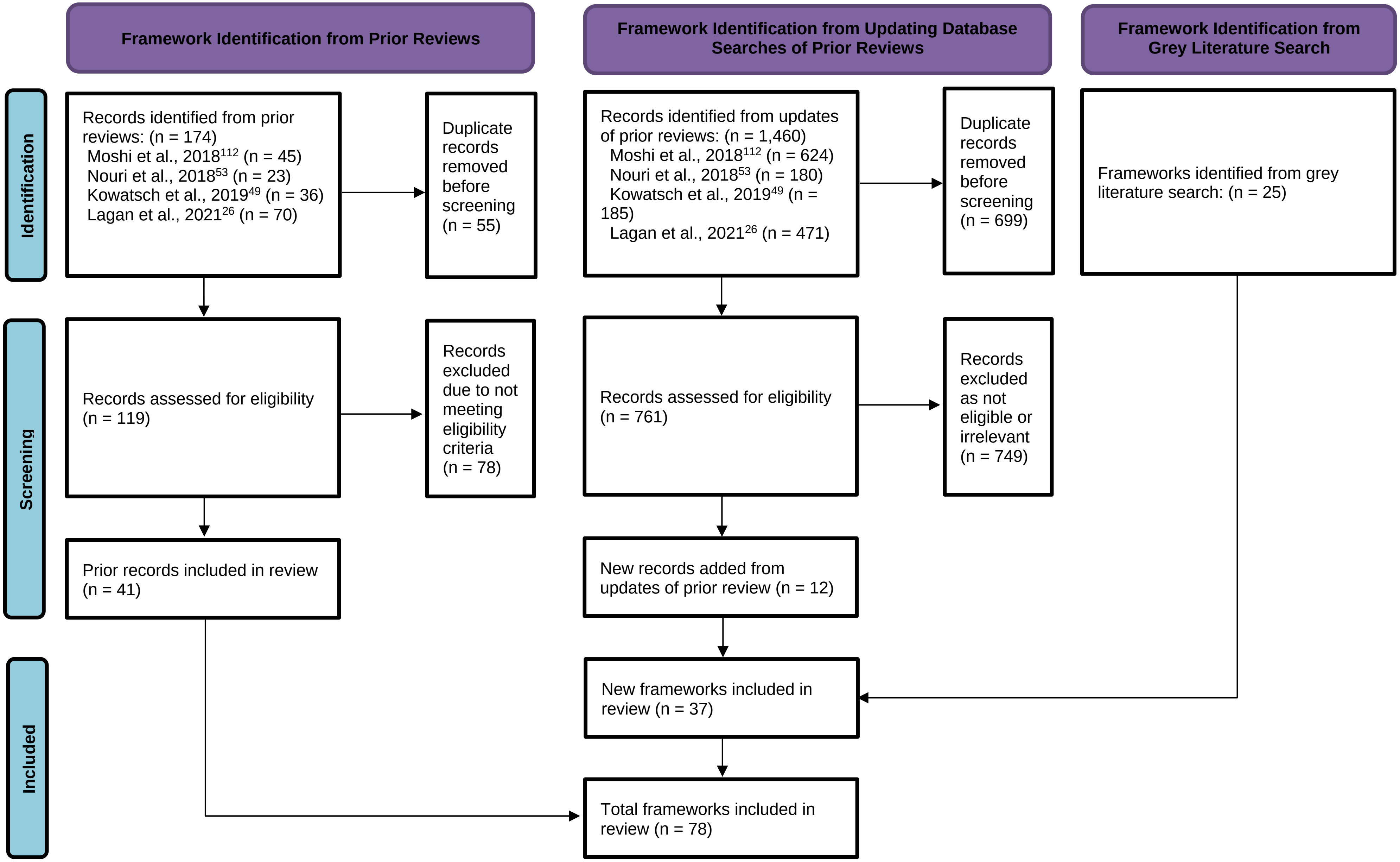

Supplementary Note 1. Framework Counts

Counts of records screened, excluded, and included are shown in Supplementary Fig. 1. A total of 78 eligible frameworks were identified. No framework met all 4 of the criteria that the workgroup believed were valuable for DHI evidence assessment. Eleven of 78 frameworks (14.1%) leverage established evidence assessment methods that were developed for non-digital interventions (eg, GRADE[1]). Just 6 frameworks (7.7%) address evidence quality criteria that may be unique to DHIs, while no framework flagged evidence quality criteria requiring increased vigilance in digital health. In just 3 of 78 frameworks (3.8%), guidelines were provided suggesting what levels of evidence quality may justify what degrees of DHI adoption. Seventy-two frameworks (92.3%), finally, addressed non-clinical assessment domains in addition to clinical evidence.

Supplementary Discussion.

Summary of literature reviewed and conclusions

The failure of any framework to meet all 4 criteria may underscore the need for a novel approach. Few frameworks explicitly recommend use of well-developed, extant methods (eg, GRADE[1]). This suggests that there may be an opportunity to improve the quality of DHI assessment in the digital health community, through consistent use of mature best practices. Failure of most frameworks to address DH-specific evidence quality considerations, and failure of any framework to address areas requiring increased vigilance, suggest that the common issues listed in Supplementary Table 2 may not be addressed adequately in prior frameworks. Finally, the rarity of evidence-to-recommendation guidelines suggests that prior frameworks may leave the evaluator without clear guidance regarding practical implications of assessment findings. For example, if a prior framework is applied and yields "moderate evidence" supporting safety and effectiveness, it may be unclear what degree of adoption is appropriate. Again, this underscores the need for novel approaches to evidence assessment in digital health.

Updating Process for the Evidence DEFINED Framework

Given the rapid evolution occurring in digital health, Evidence DEFINED will be updated every 6-12 months. Feasibility is a priority in the design of this process. A public suggestion form will be posted through the website of the Digital Medicine Society (DiMe) no more than 3 months after publication of the Framework. This will

mimic a simple form[113] posted previously on a Digital Medicine Society website[114] to gather public feedback for a prior, similar initiative.[115] Availability of the form will be announced to DiMe members and through public channels.

For each update cycle, the text of form submissions will be deidentified and compiled in a single document. Leadership from DiMe and the Evidence DEFINED Workgroup will summarize each suggestion proposed, and share compiled information with the Workgroup for feedback. Live Workgroup meetings may be convened, as needed, to address any complex suggestions. Subsequently, the following materials will be revised, as appropriate, to reflect approved updates: Evidence DEFINED Overview and Quick Start Guide (Figure 1), Supplementary Checklist of Evidence Quality Criteria for Digital Health Interventions (Supplementary Table 2), and Evidence-to-Recommendation Guidelines (Table 2). Updated Evidence DEFINED materials will be posted on DiMe's website.

Updating cycles will continue for two years. DiMe and Workgroup leadership will then decide whether or not to continue updating the Framework. This will depend on the frequency of public suggestions and resource availability.

Supplementary References.

1. Guyatt, G. H. *et al.* GRADE: an emerging consensus on rating quality of evidence and strength of recommendations. *BMJ* **336**, 924–926 (2008).

2. Mathews, S. The 6-step process for evaluating clinical impact. https://www.digitaldifferential.com/post/the-6-step-process-for-evaluating-clinical-impact (2022).

3. Digital Therapeutics Alliance. Setting the Stage for a Fit-For-Purpose DTx Evidentiary Standard. https://dtxalliance.org/wp-content/uploads/2022/12/DTA-Clinical-Evidence-Paper_12.22.pdf (2022).

4. Day, S., Shah, V., Kaganoff, S., Powelson, S. & Mathews, S. C. Assessing the clinical robustness of digital health startups: cross-sectional observational analysis. *J Med Internet Res* **24**, e37677 (2022).

5. Tarricone, R., Petracca, F., Cucciniello, M. & Ciani, O. Recommendations for developing a lifecycle, multidimensional assessment framework for mobile medical apps. *Health Econ* **31 Suppl 1**, 73–97 (2022).

6. Wagneur, N. *et al.* Assessing a new prescreening score for the simplified evaluation of the clinical quality and relevance of eHealth apps: instrument validation study. *J Med Internet Res* **24**, e39590 (2022).

7. Anxiety and Depression Association of America. Mental health apps | Anxiety and Depression Association of America. https://adaa.org/find-help/support/mental-health-apps (2022).

8. Organization for the Review of Care and Health Apps (ORCHA). Digital health assessment framework. https://dhealthframework.org/.

9. Digital Therapeutics Alliance. *DTx value assessment and integration guide 2.0* https://dtxalliance.org/advancing-dtx/dtx-value-guide/ (2022).

10. Mackey, R., Gleason, A. & Ciulla, R. A novel method for evaluating mobile apps (App Rating Inventory): development study. *JMIR mHealth and uHealth* **10**, e32643 (2022).

11. Agency for Healthcare Research and Quality. Evaluation of mental health mobile applications. https://effectivehealthcare.ahrq.gov/sites/default/files/product/pdf/mental-health-mobile-apps-tech-brief.pdf (2022).

12. Varshney, U., Singh, N., Bourgeois, A. G. & Dube, S. R. Review, Assess, Classify, and Evaluate (RACE): a framework for studying m-health apps and its application for opioid apps. *J Am Med Inform Assoc* **29**, 520–535 (2022).

13. National Library of Medicine, NIH. Evaluating mobile apps. https://omed.osteopathic.org/wp-content/uploads/2017/10/Sat-6c-Nguyen-Handout-3-Mobile-Health-App-Evaluation-Checklist.pdf (2016).

14. Cook, V. E., Ellis, A. K. & Hildebrand, K. J. Mobile health applications in clinical practice: pearls, pitfalls, and key considerations. *Ann Allergy Asthma Immunol* **117**, 143–149 (2016).

15. IQVIA. Digital health trends 2021: innovation, evidence, regulation, and adoption. https://www.iqvia.com/insights/the-iqvia-institute/reports/digital-health-trends-2021 (2021).

16. IQVIA. AppScript | discover, deliver & track digital health. https://www.appscript.net/score-details (2021).

17. American Medical Association. Return on health: moving beyond dollars and cents in realizing the value of virtual care. https://www.ama-assn.org/system/files/2021-05/ama-return-on-health-report-may-2021.pdf (2021).

18. Hensher, M. *et al.* Scoping review: Development and assessment of evaluation frameworks of mobile health apps for recommendations to consumers. *J Am Med Inform Assoc* **28**, 1318–1329 (2021).

19. International Organization for Standardization (ISO). IEC International Standard 82304-2: health software - health and wellness apps - quality and reliability. https://www.iso.org/obp/ui/#iso:std:iso:ts:82304:-2:ed-1:v1:en (2021).

20. Roberts, A. E. *et al.* Evaluating the quality and safety of health-related apps and e-tools: Adapting the Mobile App Rating Scale and developing a quality assurance protocol. *Internet Interv* **24**, 100379 (2021).

21. Wu, K.-L. *et al.* Characteristics and quality of mobile apps containing prenatal genetic testing information: systematic app store search and assessment. *JMIR Mhealth Uhealth* **9**, e30404 (2021).

22. Validation Institute. *Vendor evaluation: overview of this multi-part series* https://validationinstitute.com/wp-content/uploads/2021/04/Vendor-Evaluation_-Overview-of-this-Multi-Part-Series.pdf.

23. U.S. Department of Veterans Affairs. Value-driven framework for evaluating healthcare innovations. https://www.va.gov/INNOVATIONECOSYSTEM/assets/documents/ExecutiveReport_ValueDrivenFramework.pdf (2021).

24. Biswas, M. *et al.* ACCU3RATE: A mobile health application rating scale based on user reviews. *PLoS One* **16**, e0258050 (2021).

25. NHSX. Designing and assessing digital health services. https://www.nhsx.nhs.uk/key-tools-and-info/designing-and-building-products-and-services/.

26. Lagan, S., Sandler, L. & Torous, J. Evaluating evaluation frameworks: a scoping review of frameworks for assessing health apps. *BMJ Open* **11**, e047001 (2021).

27. Healthcare Information and Management Systems Society (HIMSS). HIMSS Digital Health Indicator Information Sheet. https://www.himssanalytics.org/himss-digital-health-indicator-information-sheet (2020).

28. Perakslis, E. & Ginsburg, G. S. Digital health-The need to assess benefits, risks, and value. *JAMA* (2020) doi:10.1001/jama.2020.22919.

29. O'Rourke, T., Pryss, R., Schlee, W. & Probst, T. Development of a multidimensional app-quality assessment tool for health-related apps (AQUA). *Digit Psych* **1**, 13–23 (2020).

30. Levine, D. M. *et al.* Design and testing of a mobile health application rating tool. *npj Digital Medicine* **3**, 1–7 (2020).

31. Ondersma, S. J. & Walters, S. T. Clinician's guide to evaluating and developing eHealth interventions for mental health. *PRCP* **2**, 26–33 (2020).

32. Camacho, E. *et al.* Technology evaluation and assessment criteria for health apps (TEACH-Apps): pilot study. *J Med Internet Res* **22**, e18346 (2020).

33. Quintana, Y. A framework for evaluation of mobile apps for youth mental health. https://bp-net.ca/wp-content/uploads/2020/11/A-Framework-for-Evaluation-of-Mobile-Apps-for-Youth-Mental-Health_May-20....pdf (2020).

34. German Federal Ministry of Health. The fast-track process for digital health applications (DiGA) according to section 139e SGB V. https://www.bfarm.de/SharedDocs/Downloads/EN/MedicalDevices/DiGA_Guide.pdf?__blob=publicationFile (2020).

35. Moshi, M. R., Tooher, R. & Merlin, T. Development of a health technology assessment module for evaluating mobile medical applications. *Int J Technol Assess Health Care* **36**, 252–261 (2020).

36. Psihogios, A. M., Stiles-Shields, C. & Neary, M. The needle in the haystack: identifying credible mobile health apps for pediatric populations during a pandemic and beyond. *J Pediatr Psychol* **45**, 1106–1113 (2020).

37. Noee, M., Akbari Sari, A., Olyaeemanesh, A. & Mobinizadeh, M. Prioritizing the potential applications of mobile-health in the Iranian health system. *J Res Health Sci* **20**, e00473 (2020).

38. Australian Commission on Safety and Quality in Healthcare. National safety and quality digital mental health standards. https://www.safetyandquality.gov.au/sites/default/files/2020-11/National%20Safety%20and%20Quality%20Digital%20Mental%20Health%20Standards%20%282%29.pdf (2020).

39. Nebeker, C., Bartlett Ellis, R. J. & Torous, J. Development of a decision-making checklist tool to support technology selection in digital health research. *Transl Behav Med* **10**, 1004–1015 (2020).

40. Vokinger, K. N., Nittas, V., Witt, C. M., Fabrikant, S. I. & von Wyl, V. Digital health and the COVID-19 epidemic: an assessment framework for apps from an epidemiological and legal perspective. *Swiss Medical Weekly* **150**, w20282 (2020).

41. Dawson, R. M. *et al.* What makes a good health "app"? Identifying the strengths and limitations of existing mobile application evaluation tools. *Nurs Inq* **27**, e12333 (2020).

42. National Institute for Health and Care Excellence (NICE). Evidence standards framework for digital health technologies. https://www.nice.org.uk/corporate/ecd7/resources/evidence-standards-framework-for-digital-health-technologies-pdf-1124017457605 (2021).

43. American Psychiatric Association. The App Evaluation Model. https://www.psychiatry.org/psychiatrists/practice/mental-health-apps/the-app-evaluation-model (2021).

44. NHS Digital. *Digital Assessment Questions V2.2*. https://confluence.hl7.org/download/attachments/64126978/WP12%20app%20checkers%20-%20NHS%20DAQ%202.2.pdf?version=1&modificationDate=1566977611627&api=v2 (2019).

45. Xcertia. Xcertia mHealth app guidelines. https://www.himss.org/sites/hde/files/media/file/2020/04/17/xcertia-guidelines-2019-final.pdf (2019).

46. Torous, J. *et al.* Towards a consensus around standards for smartphone apps and digital mental health. *World Psychiatry* **18**, 97–98 (2019).

47. Haverinen, J. *et al.* Digi-HTA: Health technology assessment framework for digital healthcare services. *Finnish Journal of eHealth and eWelfare* **11**, 326–341 (2019).

48. Mathews, S. C. *et al.* Digital health: a path to validation. *NPJ Digit Med* **2**, 38 (2019).

49. Kowatsch, T., Otto, L., Harperink, S., Cotti, A. & Schlieter, H. A design and evaluation framework for digital health interventions. *it - Information Technology* **61**, 253–263 (2019).

50. Henson, P., David, G., Albright, K. & Torous, J. Deriving a practical framework for the evaluation of health apps. *Lancet Digit Health* **1**, e52–e54 (2019).

51. Wykes, T. & Schueller, S. Why reviewing apps is not enough: Transparency for Trust (T4T) principles of responsible health app marketplaces. *J Med Internet Res* **21**, e12390 (2019).

52. Torous, J. B. *et al.* A hierarchical framework for evaluation and informed decision making regarding smartphone apps for clinical care. *Psychiatr Serv* **69**, 498–500 (2018).

53. Nouri, R., R Niakan Kalhori, S., Ghazisaeedi, M., Marchand, G. & Yasini, M. Criteria for assessing the quality of mHealth apps: a systematic review. *J Am Med Inform Assoc* **25**, 1089–1098 (2018).

54. Evans, K., Donelan, J., Rennick-Egglestone, S., Cox, S. & Kuipers, Y. Review of mobile apps for women with anxiety in pregnancy: maternity care professionals' guide to locating and assessing anxiety apps. *J Med Internet Res* **24**, e31831 (2022).

55. U.S. Department of Defense. U.S. Department of Defense Mobile Health Practice Guide, Fourth Edition. https://telemedicine.arizona.edu/sites/default/files/DoD%20Mobile%20Health%20Practice%20Guide-Fourth%20Edition.pdf (2018).

56. Mental Health Commission of Canada. Toolkit for e-mental health implementation. https://mentalhealthcommission.ca/wp-content/uploads/2021/05/E_Mental_Health_Implementation_Toolkit_2018_eng.pdf (2018).

57. Zelmer, J. *et al.* An assessment framework for e-Mental health apps in Canada: results of a modified Delphi process. *JMIR mHealth and uHealth* **6**, e10016 (2018).

58. Wyatt, J. C. How can clinicians, specialty societies and others evaluate and improve the quality of apps for patient use? *BMC Medicine* **16**, 225 (2018).

59. Betton, V. *et al.* Framework for the effectiveness evaluation of mobile (mental) health tools. https://s3-eu-west-1.amazonaws.com/assets.wearemhabitat.com/Framework-for-the-effectiveness-evaluation-of-mobile-mental-health-tools.pdf (2017).

60. Baumel, A., Faber, K., Mathur, N., Kane, J. M. & Muench, F. Enlight: a comprehensive quality and therapeutic potential evaluation tool for mobile and web-based eHealth interventions. *J Med Internet Res* **19**, e7270 (2017).

61. Leigh, S., Ouyang, J. & Mimnagh, C. Effective? Engaging? Secure? Applying the ORCHA-24 framework to evaluate apps for chronic insomnia disorder. *Evid Based Ment Health* **20**, e20 (2017).

62. Royston, G. Rapid methods to assess the potential impact of digital health interventions, and their application to low resource settings. in *Proceedings of the*

2017 International Conference on Digital Health 82–89 (Association for Computing Machinery, 2017). doi:10.1145/3079452.3079466.

63. Basilico, A., Marceglia, S., Bonacina, S. & Pinciroli, F. Advising patients on selecting trustful apps for diabetes self-care. *Comput Biol Med* **71**, 86–96 (2016).
64. Murray, E. *et al.* Evaluating digital health interventions: key questions and approaches. *Am J Prev Med* **51**, 843–851 (2016).
65. Agarwal, S. *et al.* Guidelines for reporting of health interventions using mobile phones: mobile health (mHealth) evidence reporting and assessment (mERA) checklist. *BMJ* **352**, i1174 (2016).
66. Haute Autorite De Sante. Good practice guidelines on health apps and smart devices. https://www.has-sante.fr/upload/docs/application/pdf/2017-03/dir1/good_practice_guidelines_on_health_apps_and_smart_devices_mobile_health_or_mhealth.pdf (2016).
67. Maheu M, Nicolucci V, Pulier M, Wall K, Frye J, Hudlicka E. The Interactive Mobile App Review Toolkit (IMART): a clinical practice-oriented system. *J Technol Behav Sci* **1**, 3–15 (2016).
68. Drincic, A., Prahalad, P., Greenwood, D. & Klonoff, D. C. Evidence-based mobile medical applications in diabetes. *Endocrinol Metab Clin North Am* **45**, 943–965 (2016).
69. Stoyanov, S. R. *et al.* Mobile App Rating Scale: a new tool for assessing the quality of health mobile apps. *JMIR mHealth and uHealth* **3**, e3422 (2015).

70. British Standards Institute. PAS 277:2015: Health and wellness apps - quality criteria across the life cycle - Code of practice. (2015).

71. Wyatt, J. C. *et al.* What makes a good clinical app? Introducing the RCP Health Informatics Unit checklist. *Clin Med (Lond)* **15**, 519–521 (2015).

72. McMillan, B., Hickey, E., Patel, M. G. & Mitchell, C. Quality assessment of a sample of mobile app-based health behavior change interventions using a tool based on the National Institute of Health and Care Excellence behavior change guidance. *Patient Educ Couns* **99**, 429–435 (2016).

73. Chan, S., Torous, J., Hinton, L. & Yellowlees, P. Towards a framework for evaluating mobile mental health apps. *Telemed J E Health* **21**, 1038–1041 (2015).

74. Boudreaux, E. D. *et al.* Evaluating and selecting mobile health apps: strategies for healthcare providers and healthcare organizations. *Transl Behav Med* **4**, 363–371 (2014).

75. Aungst, T. D., Clauson, K. A., Misra, S., Lewis, T. L. & Husain, I. How to identify, assess and utilise mobile medical applications in clinical practice. *Int J Clin Pract* **68**, 155–162 (2014).

76. Hanrahan, C., Aungst, T. D. & Cole, S. *Evaluating Mobile Medical Applications*. (American Society of Health-System Pharmacists, 2014).

77. PsyberGuide. One Mind PsyberGuide. https://onemindpsyberguide.org/apps-workplace/.

78. Albrecht, U.-V., Von Jan, U. & Pramann, O. Standard reporting for medical apps. *Stud Health Technol Inform* **190**, 201–203 (2013).

79. Albrecht, U.-V., von Jan, U., Jungnickel, T. & Pramann, O. App-synopsis - standard reporting for medical apps. in *Social Media and Mobile Technologies for Healthcare* 94–108 (Medical Information Science Reference, 2013).

80. Beatty, A. L., Fukuoka, Y. & Whooley, M. A. Using mobile technology for cardiac rehabilitation: a review and framework for development and evaluation. *J Am Heart Assoc* **2**, (2013).

81. Khoja, S., Durrani, H., Scott, R. E., Sajwani, A. & Piryani, U. Conceptual framework for development of comprehensive e-health evaluation tool. *Telemed J E Health* **19**, 48–53 (2013).

82. Kidholm, K. *et al.* A model for assessment of telemedicine applications: mast. *Int J Technol Assess Health Care* **28**, 44–51 (2012).

83. Eysenbach, G. & CONSORT-EHEALTH Group. CONSORT-EHEALTH: improving and standardizing evaluation reports of Web-based and mobile health interventions. *J Med Internet Res* **13**, e126 (2011).

84. Silberman, J., Sarlati, S., Kaur, M. & Bokhari, W. Chapter 23 - Outcomes assessment for digital health interventions in diabetes: a payer perspective. in *Diabetes Digital Health and Telehealth* (eds. Klonoff, D. C., Kerr, D. & Weitzman, E. R.) 291–304 (Academic Press, 2022). doi:10.1016/B978-0-323-90557-2.00023-6.

85. Twenge, J. M. & Campbell, W. K. Associations between screen time and lower psychological well-being among children and adolescents: Evidence from a population-based study. *Prev Med Rep* **12**, 271–283 (2018).

86. Twenge, J. M., Martin, G. N. & Campbell, W. K. Decreases in psychological well-being among American adolescents after 2012 and links to screen time during the rise of smartphone technology. *Emotion* **18**, 765–780 (2018).

87. Cheung, M.-C., Lai, J. S. K. & Yip, J. Influences of Smartphone and Computer Use on Health-Related Quality of Life of Early Adolescents. *Int J Environ Res Public Health* **19**, 2100 (2022).

88. Brailovskaia, J., Ströse, F., Schillack, H. & Margraf, J. Less Facebook use – More well-being and a healthier lifestyle? An experimental intervention study. *Computers in Human Behavior* **108**, 106332 (2020).

89. Twenge, J. M. Why increases in adolescent depression may be linked to the technological environment. *Curr Opin Psychol* **32**, 89–94 (2020).

90. Wacks, Y. & Weinstein, A. M. Excessive smartphone use is associated with health problems in adolescents and young adults. *Front Psychiatry* **12**, 669042 (2021).

91. Freedland, K. E., Mohr, D. C., Davidson, K. W. & Schwartz, J. E. Usual and unusual care: existing practice control groups in randomized controlled trials of behavioral interventions. *Psychosom Med* **73**, 323–335 (2011).

92. Lin, L. Bias caused by sampling error in meta-analysis with small sample sizes. *PLoS One* **13**, e0204056 (2018).

93. Goldberg, S. B., Bolt, D. M. & Davidson, R. J. Data missing not at random in mobile health research: assessment of the problem and a case for sensitivity analyses. *J Med Internet Res* **23**, e26749 (2021).

94. Eysenbach, G. The law of attrition. *J Med Internet Res* **7**, e402 (2005).

95. National Research Council (US) Panel on Handling Missing Data in Clinical Trials. The prevention and treatment of missing data in clinical trials. http://www.ncbi.nlm.nih.gov/books/NBK209904/ (2010).

96. Little R, Rubin R. *Statistical analysis with missing data*. (Wiley, 2002).

97. Little, R. J. *et al.* The prevention and treatment of missing data in clinical trials. *N Engl J Med* **367**, 1355–1360 (2012).

98. Straus S, Glasziou P, Richardson W, Haynes RB. *Evidence-based medicine: How to practice and teach EBM.* (Elsevier, 2019).

99. Fernández-García, M. P., Vallejo-Seco, G., Livácic-Rojas, P. & Tuero-Herrero, E. The (ir)responsibility of (under)estimating missing data. *Frontiers in Psychology* **9**, (2018).

100. Li, T. *et al.* Standards in the prevention and handling of missing data for patient centered outcomes research – a systematic review and expert consensus. *J Clin Epidemiol* **67**, 15–32 (2014).

101. Reichardt CS. Randomized Experiments. in *Quasi-Experimentation* 45–94 (Guilford Press, 2019).

102. Jakobsen, J. C., Gluud, C., Wetterslev, J. & Winkel, P. When and how should multiple imputation be used for handling missing data in randomised clinical trials – a practical guide with flowcharts. *BMC Medical Research Methodology* **17**, 162 (2017).

103. ICMJE. Recommendations | clinical trials. https://www.icmje.org/recommendations/browse/publishing-and-editorial-issues/clinical-trial-registration.html (2022).

104. National Institutes of Health. Summary table of HHS/NIH initiatives to enhance availability of clinical trial Information. https://www.nih.gov/news-events/summary-table-hhs-nih-initiatives-enhance-availability-clinical-trial-information (2016).

105. Eysenbach, G. Tackling publication bias and selective reporting in health informatics research: register your eHealth trials in the International eHealth Studies Registry. *J Med Internet Res* **6**, e35 (2004).

106. Sedhom, R., McShea, M. J., Cohen, A. B., Webster, J. A. & Mathews, S. C. Mobile app validation: a digital health scorecard approach. *npj Digit. Med.* **4**, 1–8 (2021).

107. Grudniewicz, A. *et al.* Predatory journals: no definition, no defence. *Nature* **576**, 210–212 (2019).

108. Shrank, W. H., Patrick, A. R. & Alan Brookhart, M. Healthy user and related biases in observational studies of preventive interventions: a primer for physicians. *J Gen Intern Med* **26**, 546–550 (2011).

109. Morawski, K. *et al.* Association of a smartphone application with medication adherence and blood pressure control: the MedISAFE-BP randomized clinical trial. *JAMA Intern Med* **178**, 802–809 (2018).

110. Schueller, S. M. & Torous, J. Scaling evidence-based treatments through digital mental health. *Am Psychol* **75**, 1093–1104 (2020).

111. GRADE Working Group. *5.2.2 Inconsistency of results, GRADE Handbook* https://gdt.gradepro.org/app/handbook/handbook.html#h.g2dqzi9je57e (2013).

112. Moshi, M. R., Tooher, R. & Merlin, T. Suitability of current evaluation frameworks for use in the health technology assessment of mobile medical applications: A systematic review. *Int J Technol Assess Health Care* **34**, 464–475 (2018).

113. Digital Medicine Society (DiMe). Share your feedback on the EVIDENCE checklist. https://docs.google.com/forms/d/e/1FAIpQLScfknZaR5T3xDxZGRyANOWsti1BxjnD2MU2x-qe16QmeO_JLQ/viewform?usp=embed_facebook.

114. Digital Medicine Society. EVIDENCE Checklist. *Digital Medicine Society (DiMe)* https://www.dimesociety.org/access-resources/evidence/.

115. Manta, C. *et al.* EVIDENCE publication checklist for studies evaluating connected sensor technologies: explanation and elaboration. *Digit Biomark* **5**, 127–147 (2021).
